# Requirements and Metabolism for Calcium, Phosphorus and Vitamin D_3_ in the Growing–Furring Blue Foxes

**DOI:** 10.3390/ani12202776

**Published:** 2022-10-14

**Authors:** Ting Li, Zhiheng Du, Yinan Xu, Xiujuan Bai, Guangyu Li

**Affiliations:** 1 College of Animal Sciences and Technology, Northeast Agricultural University, Harbin 150030, China; 2 College of Animal Science and Technology, Qingdao Agricultural University, Qingdao 266109, China

**Keywords:** blue fox, calcium, growth performance, nutrient digestibility, vitamin D_3_

## Abstract

**Simple Summary:**

The nutritional needs of mink and foxes have long been clear, not specifically for blue foxes. With the development of technology, the present study aimed to study the effect of dietary calcium, phosphorus, and vitamin D_3_ supplementation levels on the growth performance, nutrient digestibility, and serum biochemical indicators of the growing hairy blue fox. The results showed that Ca (0.8–1.2%) and vitamin D_3_ (1327 IU·kg^−1^) doses have important effects on the growth performance and nutrient digestibility of growing blue foxes and can reduce fecal nitrogen and fecal phosphorus by improving the utilization of protein and phosphorus.

**Abstract:**

A 3 × 3 factorial experiment was conducted to investigate the influence of dietary calcium, phosphorus, and vitamin D_3_ (VD_3_) supplement levels on the growth performance, nutrient digestibility, and serum biochemical indices of growing-furring blue foxes. One hundred and thirty-five 120-day-old male blue foxes were randomly allocated into nine groups. The nine treatment diets were supplemented with 0%, 0.4%, or 0.8% Ca, and 1000, 2000, or 4000 IU·kg^−1^ VD_3_. The base diet contained 0.8% Ca and 327 IU·kg^−1^ VD_3_. The dietary calcium level had a significant effect on the average daily gain (ADG) of blue foxes at 121 to 135 days of age and 136 to 150 days (*p* < 0.05). The ADG of blue foxes at 121 to 135 days of age was significantly decreased by VD_3_ level (*p* < 0.05). The Ca dosage decreased the nutrient digestibility (*p* < 0.05). The Ca dosage increased the fecal Ca and P and decreased the P digestibility (*p* < 0.05). Interactions were found between the Ca and VD_3_ levels, which affected the digestibility of Ca and P (*p* < 0.05). In conclusion, this research determined the suitable doses of Ca and VD_3_ for growing-furring blue foxes.

## 1. Introduction

Calcium (Ca) and phosphorus (P) are critically important for many body functions. Most of the Ca in the body is located within the skeleton and teeth, and along with phosphate anions, provides structural strength and hardness in the skeleton and teeth [1]. A deficiency of Ca cause various diseases, such as osteoporosis and chondropathy [2]. Therefore, the regulation of their plasma concentrations is tightly controlled by the concerted actions of reabsorption/excretion in the kidneys, absorption in the intestines, and exchanges from bone, which is the major reservoir for Ca and P in the body [3]. In addition, the proportion of Ca/P in the diet affects the absorption of Ca and P. Jorgensen indicated that when minks were fed 100 IU/mink/day, a suitable Ca/P ratio was in the range of 0.75–1.7 [4].

Vitamin D_3_ (VD_3_) is important for the mineralization of bone tissue, and VD_3_ deficiency is a worldwide epidemic and a factor in multifactorial causes of nonvertebral and hip fractures, falls, and muscle power loss [5]. Experimental studies by Smith and Barnes indicated that with an optimum Ca/P ratio of 1/1, rickets was not produced, even with an experimental ratio that was relatively low in VD_3_ [6].

Blue foxes (*Alopex lagopus*) have a great reputation in the pelt industry around the world because of their large size and high-ranking fur quality. Since the 1980s, the nutrient requirements have been established for minks and foxes but not specifically for blue foxes [7]. However, even with all factors considered, the studied data may be skewed because foxes today are larger than the small animals that were employed in these studies, and the significant body size may have altered the nutritional requirements of blue foxes for growth and fur quality [8]. Kenneth’s research explains the differences in nutrient requirements between different species of animals [9]. Although Liu et al. [10] has reported the needs for calcium, phosphorus, and VD_3_ of blue foxes in the early growth stage, the needs of calcium and phosphorus of blue foxes in the growth winter hair stage are still unknown. The objective of the present study was to evaluate the effects of dietary Ca and VD_3_ supplemental levels on the growth performance, nutrient digestibility, and serum biochemical indices in growing-furring blue foxes.

## 2. Materials and Methods

All the protocols of our experiment were approved by the Animal Welfare and Ethics Committee of Northeast Agricultural University. The experiment was performed from 12 September to 8 December 2016.

### 2.1. Experimental Design, Diets, and Sampling

One hundred and thirty-five 120-day-old male blue foxes (mean body weight ± SD, 4.03 ± 0.25 kg) were randomly assigned to 9 treatment groups with 15 blue foxes per treatment. The foxes were fed dry diets supplemented with 0%, 0.4%, or 0.8% Ca, and the diets were supplemented with 1000, 2000, or 4000 IU·kg^−1^ VD_3_. The base diet contained 0.8% Ca and 327 IU·kg^−1^ VD_3_. The composition and chemical analysis of the basal diet is shown in Table 1. The ratio of dietary Ca and P was kept constant (1.4/1.0). The experiment was preceded by a 1-week adjustment period, and the experimental period lasted 87 days. All foxes were housed individually in conventional cages in a south-row shed. The diets were supplied twice a day at 08:00 h and 15:00 h. Drinking water was freely available. All animals were weighed in the morning before feeding once every two weeks. The digestive experiment was conducted from 15 October to 17 October and lasted for 3 days. Then, eight randomly selected animals from each treatment group were housed individually in metabolic cages that permitted the separation of urine and feces [11]. Feed intake and residues were accurately recorded to calculate the ADG and G: F. Feces and urine were collected daily and stored at −20 °C until analyzed. According to the volume of urine, 10 mL sulfuric acid (10% solution) per 100 mL of urine was added to combine the nitrogen to prevent nitrogen loss. The fecal collection trays were sprayed with sulfuric acid (10% solution) once per day. Eight blue foxes were selected randomly from each group, and their blood was taken immediately at the end of the digestive experiment. The samples (10 mL) were collected in 2 separate tubes with 10 μL of procoagulant substance. The samples were transferred to the lab, where the serum was obtained by centrifuging the tubes at 2500× *g* at 4 °C for 300 s. The serum was separated from the blood, transferred into Eppendorf centrifuge tubes, and kept at −20 °C until analysis. 

### 2.2. Slaughter Traits

Eight randomly selected blue foxes from each group were pelted according to normal farming practices. Twenty to thirty blue foxes were placed in a killing box depending on the box size, and killed with gas (CO_2_) according to the requirements of the Welfare of Animals Kept for Fur Production. 

### 2.3. Analysis Method

The nutrient contents of the feed, feces, and urine were analyzed using the methods of the Association of Official Analytical Chemists (AOAC). Dry matter was quantified by drying feed or fecal samples at 105 °C to a constant weight. The fat (EE) content of the feed and feces was determined using a diethyl ether extraction–submersion method. The nitrogen in the sample was measured by a FOSS Kjeltec 8400 analyzer (Foss Electric, Sweden), and the crude protein was calculated as N × 6.25. The contents of ASH, Ca, and P were analyzed according to AOAC procedures [12]. Carbohydrate (CHO) content was calculated by subtracting the ASH, CP, and EE contents from the DM content. The ME content of the feed was calculated based on the concentrations of CP, EE, and CHO; the reference digestibility coefficients from the Danish Standard Values for individual feed; and the following ME content values per unit of digestible nutrients (KJ/g): CP, 18.8; EE, 39.8; and CHO, 17.6 [13]. The total serum biochemical indices were measured with Bradford’s method using a standard kit (Nanjing Jiancheng Biotechnology Co., Ltd., Nanjing, China) [14].

N deposition = N intake − fecal N − urinary N

Net protein utilization (NPU) (%) = N deposition/N intake × 100%

Biological value (BV) (%) = N deposition/(N intake − fecal N) × 100%

### 2.4. Statistics

All data were analyzed using the General Linear Model procedure of SAS (SAS Inst. Inc., Cary, NC, USA) as appropriate for a randomized complete block design two-way ANOVA [15]. Differences among all groups were tested using Duncan’s test. Differences were considered significant at *p* < 0.05.

## 3. Results

### 3.1. Growth Performance

The dietary calcium level had a significant effect on the ADG of blue foxes at 121 to 135 days of age and 136 to 150 days (*p* < 0.05, Table 2). The ADG of blue foxes at 121 to 135 days of age was significantly decreased by VD_3_ level (*p* < 0.05). The ADG of the low-level Ca and the low-level VD_3_ was the highest. Interactions between the Ca and VD_3_ levels affected the ADG at 121 to 135 days of age (*p* < 0.05).

### 3.2. Digestibility

The Ca dosage decreased the DM and CHO digestibility (*p* < 0.05, Table 3). The EE digestibility initially increased and subsequently decreased with increasing Ca levels (*p* < 0.05). The VD_3_ dosage had a significant effect on the EE digestibility (*p* < 0.05). The lowest EE was observed in the mid-level VD_3_ group. Significant interactions between the Ca and VD_3_ levels affected the digestibility of EE (*p* < 0.05).

### 3.3. N Metabolism

The Ca dose initially increased and subsequently decreased the fecal N (*p* < 0.05, Table 4). The supplemental doses of Ca linearly decreased the N intake, N deposition, NPU, and BV of protein (*p* < 0.05) but did not alter the urinary N (*p* > 0.05). The N intake and BV of protein were improved by the VD_3_ level (*p* < 0.05). Significant interactions between the Ca and VD_3_ levels affected the N intake and BV of protein (*p* < 0.05).

### 3.4. Ca and P Digestibility

The fecal Ca and P improved and the P digestibility declined as Ca increased in the diet (*p* < 0.05, Table 5). Interactions between Ca and VD_3_ levels affected the digestibility of Ca and P (*p* < 0.05). 

### 3.5. Serum Biochemical Indices

The total protein (TP) initially increased and subsequently decreased with increases in dietary VD_3_ (*p* < 0.05, Table 6). The highest TP was observed in the mid-level VD_3_ group. Interactions between Ca and VD_3_ levels affected the serum Ca (*p* < 0.05).

### 3.6. Serum Hormone 

The apparent parathyroid hormone (PTH) and calcitonin (CT) levels decreased with increasing Ca level (*p* < 0.05, Table 7). Calcitonin presented a linear (*p* < 0.05) trend with increasing levels of VD_3_. With an increase in dietary VD_3_, the 25-OH-D_3_ value initially decreased and subsequently increased (*p* < 0.05). The interaction between dietary levels of Ca and VD_3_ significantly influenced the PTH, CT, and 25-OH-D_3_.

## 4. Discussion

### 4.1. Growth Performance

This study indicated that the dietary Ca level significantly affected the final BW, ADG, and ADFI. The highest values of the final BW, ADG, and ADFI were observed in the low-level Ca group. Increasing Ca supplementation will suppress the growth of blue foxes. One experiment was conducted to determine the influence of dietary Ca concentrations (0.4, 0.6, 0.8, 1.0, 1.2, 1.4, or 1.6% of the diet) in corn–soybean meal diets fed to broiler chickens from 2 to 23 days of age, and increasing the dietary Ca concentrations elicited linear reductions in the overall growth performance [16]. Another experiment showed that body weight gain and feed intake were depressed with an increase in the level of Ca at lower levels of NPP (3 and 3.5 g·kg^−1^ diet) at 14, 28, and 42 days of age in commercial broilers, and these negative effects were alleviated by reducing the levels of Ca to the minimum tested levels [17]. When animals were fed Ca above the maximum tolerable levels over a longer period of time, a significant reduction in feed intake that affected performance was observed [18]. The interaction between separate Ca feeding and phytase supplementation on performance in broiler starters was investigated, and increasing dietary Ca concentration was found to decrease weight gain and feed intake [19]. In one experiment, high dietary Ca (24.3 versus 11.8 g·kg^−1^) reduced performance, and these results are consistent with the results of this study [20]. We speculated that a low level of Ca was adequate for the growing-furring period of blue fox.

### 4.2. Digestibility

This study has shown that an excess supplemental dose of Ca linearly decreased the digestibility of certain nutrients. Excessive concentrations of dietary Ca may impede the availability of nutrients by the formation of nonabsorbable complexes [21]. A previous experiment revealed that high inorganic P and Ca levels may have negative effects on pig performance when the positive effects of antimicrobial growth promoters are removed from weaner pig diets [22]. The EE content is associated with fur quality, and thicker subcutaneous fat corresponds to a better extension of fur. Moreover, fur animals present higher EE digestibility than other animals, and the EE digestibility has been reported to exceed 90% [23]. Calcium plays an important role in intestinal lipid digestion by increasing the lipolysis rate, and it also limits fatty acid bioaccessibility by producing insoluble Ca soaps with long-chain fatty acids under intestinal pH conditions [24]. Therefore, EE digestibility first increased and subsequently decreased with increasing Ca levels in the diet. In our study, the EE digestibility in the mid-level Ca group was the highest.

### 4.3. N Metabolism

A significant response in N metabolism was observed with the dietary Ca dose in blue foxes, and this response was consistent with the variation in growth performance. An experiment on the effects of variations in dietary Ca, available P, and protein on the performance and N utilization in broiler chickens reported that high dietary Ca (24.3 versus 11.8 g·kg^−1^) reduced performance, N digestibility, and ME [20]. Because the ADFI presented a linear response under increasing levels of Ca, a reduction in the consumption of all nutritional substances may have occurred, and this trend likely led to a decline in N intake, N deposition, NPU, and BV of protein.

### 4.4. Ca and P Digestibility

A relationship was observed between Ca absorption and body requirements. The absorption speed increased as the Ca content decreased and decreased with excess Ca. With an increase in the Ca levels, P digestibility declined. These results indicate that Ca levels meet the requirements for growth in blue foxes. Moreover, excessive Ca influences the absorption of P, which is consistent with the results of a study in pigs [25]. Pigs that were offered low and medium P diets showed a consistently lower fecal amount than pigs offered a high P diet over days 0 to 34 [22]. Ca and P have an antagonistic relationship, and increasing dietary Ca reduced P absorption and also reduced the utilization of phytate P [26,27]. Phytate P utilization is influenced by numerous factors, such as dietary Ca and P, phytase, VD_3_ and its derivatives, as well as organic acids [28,29]. Vitamin D_3_ plays an important role in regulating Ca and P absorption, deposition, and dissolution. Vitamin D_3_ increases Ca intestinal absorption by removing Ca from the gastrointestinal tract and subsequently increases phytate P utilization [26]. Interactions between the Ca and VD_3_ levels affected the digestibility of Ca and P.

### 4.5. Serum Biochemical Indices

The serum biochemical indices of normal animals could reflect normal physiological conditions and have an important connection with the body’s metabolism, nutrition, and disease. The serum TP represents the protein level in the diet and the absorption degree of dietary protein for animals. The N intake and the BV of protein increased with increasing VD_3_ levels. Therefore, the serum TP had a similar influence as the VD_3_ levels increased. However, at high VD_3_ levels, the serum TP decreased. This trend may be related to the growing-furring period of the blue foxes. Serum alkaline phosphatase (ALP) is of interest in the diagnosis of two main groups of conditions: hepatobiliary disease and bone disease associated with increased osteoblastic activity. A study on the response of plasma ALP, PTH, blood, and bone minerals to Ca intake in fowl showed that increasing amounts of ALP corresponded to inadequate bone mineralization [30]. The serum ALP level could be used as an auxiliary index to diagnose rickets because serum ALP increases sharply when rickets occurs [31]. Serum ALP can also be used as an important test indicator in a Ca and P feeding experiment or balance test to assess the appropriate levels of Ca and P in the diet [32].

### 4.6. Serum Hormone

Serum Ca is tightly regulated by the actions of PTH, VD_3_, and CT, and this regulation is critical for normal cell function, neural transmission, membrane stability, bone structure, blood coagulation, and intracellular signaling. The interactions among PTH, VD_3_, and CT maintain normal serum Ca levels [33]. Because of regulation by Ca sensing receptors located in the parathyroid gland, the concentration of extracellular Ca is maintained by intestinal absorption, kidney reabsorption, and bone resorption/formation [34,35]. Vitamin D_3_ and Ca metabolism are complex and interrelated, and they are also tightly controlled via feedback loops established to conserve Ca homeostasis. When plasma Ca is elevated, PTH is inhibited, the kidneys excrete Ca, and CT is excreted from the thyroid to induce Ca accretion in bone. When plasma Ca is below normal, the parathyroid releases PTH, which causes bone resorption of Ca and P, and then the kidneys activate vitamin D_3_, which in turn increases the absorption of Ca and P [36].

## 5. Conclusions

In conclusion, the results indicate that the doses of Ca (0.8–1.2%) and VD_3_ (1327 IU·kg^−1^) had an important influence on the growth performance and nutrient digestibility of growing-furring blue foxes and could reduce the fecal N and fecal P by improving protein and P utilization.

## Figures and Tables

**Table 1:** Composition of the 9 categories of diets provided to 120-day-old maturing and furring blue foxes (air-dry basis).

Items	Groups								
	Ⅰ	Ⅱ	Ⅲ	Ⅳ	Ⅴ	Ⅵ	Ⅶ	Ⅷ	Ⅸ
Ingredients (%)									
Extruded corn	39.8	39.8	39.8	38.2	38.2	38.2	36.3	36.3	36.3
Soybean meal	20.0	20.0	20.0	20.0	20.0	20.0	20.0	20.0	20.0
DDGS	5.0	5.0	5.0	5.0	5.0	5.0	5.0	5.0	5.0
Corn protein meal	11.3	11.3	11.3	11.5	11.5	11.5	12.0	12.0	12.0
Fish meal	10.0	10.0	10.0	10.0	10.0	10.0	10.0	10.0	10.0
Chicken meal	2.0	2.0	2.0	2.0	2.0	2.0	2.0	2.0	2.0
CaHPO_4_	0.0	0.0	0.0	1.4	1.4	1.4	2.8	2.8	2.8
Limestone	0.9	0.9	0.9	0.9	0.9	0.9	0.9	0.9	0.9
Soybean oil	10.0	10.0	10.0	10.0	10.0	10.0	10.0	10.0	10.0
Premix ^a^	1.0	1.0	1.0	1.0	1.0	1.0	1.0	1.0	1.0
Total	100.0	100.0	100.0	100.0	100.0	100.0	100.0	100.0	100.0
Chemical composition of diet									
ME ^b^ (MJ kg^−1^)	14.1	14.1	14.1	14.0	14.0	14.0	14.0	14.0	14.0
CP (%)	30.2	30.2	30.2	30.2	30.2	30.2	30.3	30.3	30.3
Lys (%)	1.4	1.4	1.4	1.4	1.4	1.4	1.4	1.4	1.4
Met (%)	0.8	0.8	0.8	0.8	0.8	0.8	0.8	0.8	0.8
EE ^c^ (%)	10.8	10.8	10.8	10.6	10.6	10.6	10.7	10.7	10.7
Ca (%)	0.8	0.8	0.8	1.2	1.2	1.2	1.6	1.6	1.6
Total P (%)	0.6	0.6	0.6	0.9	0.9	0.9	1.1	1.1	1.1
Ca/P	1.4	1.4	1.4	1.4	1.4	1.4	1.4	1.4	1.4
VD_3_ ^d^ (IU kg^−1^)	1327	2327	4327	1327	2327	4327	1327	2327	4327

^a^ Nutrient level/kg of diet: Fe 80 mg; Zn 60 mg; Mn 15 mg; Cu 10 mg; I 0.5 mg; Se 0.2 mg; Co 0.3 mg; vitamin A 10,000 IU; vitamin E 60 mg; vitamin K3 1.6 mg; vitamin B1 20 mg; vitamin B2 10 mg; vitamin B6 10 mg; vitamin B12 0.1 mg; niacin 40 mg; pantothenic acid 20 mg; folic acid 1 mg; biotin 0.5 mg; vitamin C 120 mg; and choline 400 mg. ^b^ ME = values were calculated, whereas remaining nutrient values were measured. ^c^ EE = ether extract. ^d^ VD_3_ = vitamin D_3_.

**Table 2:** The outcome of Ca and VD_3_ diet supplementation on ADG of maturing and furring blue foxes.

Items	Ca Level (%)			VD_3_ * Level (IU·kg^−1^)			SEM	*p*-Value		
	0	0.4	0.8	1000	2000	4000		Ca Level	VD_3_ Level	Ca × VD_3_ Interaction
106–120 days (kg)	0.72	0.77	0.72	0.75	0.73	0.73	0.024	0.310	0.858	0.075
121–135 days (kg)	0.45 ^a^	0.34 ^b^	0.40 ^ab^	0.45 ^a^	0.41 ^a^	0.34 ^b^	0.013	0.002	0.001	<0.001
136–150 days (kg)	0.43 ^a^	0.32 ^b^	0.34 ^b^	0.38	0.35	0.36	0.012	<0.001	0.510	0.361

Values with dissimilar lowercase letters in a given row represent a significant difference (*p* < 0.05). Similar letters, or absence of letters, reflect no significant change (*p* > 0.05). VD_3_ * = vitamin D_3_.

**Table 3:** The outcome of Ca and VD_3_ diet supplementation on nutrient digestibility in maturing and furring blue foxes.

Items	Ca Level (%)			VD_3_ * Level (IU·kg^−1^)			SEM	*p*-Value		
	0	0.4	0.8	1000	2000	4000		Ca Level	VD_3_ Level	Ca × VD_3_ Interaction
DM digestibility (%)	69.20 ^a^	67.28 ^b^	64.85 ^c^	67.92	66.51	66.86	9.092	<0.001	0.257	0.562
CP digestibility (%)	69.98	68.12	68.05	68.70	68.28	69.22	15.914	0.176	0.726	0.825
EE ** digestibility (%)	90.48 ^b^	91.94 ^a^	91.52 ^a^	91.84 ^a^	90.34 ^b^	91.33 ^a^	1.989	0.007	0.023	0.002
CHO *** digestibility (%)	71.42 ^a^	70.69 ^ab^	68.98 ^b^	71.64	69.89	69.52	13.835	0.007	0.119	0.265

Values with dissimilar lowercase letters in a given row represent a significant difference (*p* < 0.05). Similar letters, or absence of letters, reflect no significant change (*p* > 0.05). VD_3_ * = vitamin D_3_. EE * = ether extract. CHO *** = carbohydrate.

**Table 4:** The outcome of Ca and VD_3_ diet supplementation on N metabolism in maturing and furring blue foxes.

Items	Ca Level (%)			VD_3_ * Level (IU·kg^−1^)			SEM	*p*-Value		
	0	0.4	0.8	1000	2000	4000		Ca Level	VD_3_ Level	Ca × VD_3_ Interaction
N intake (g d^−1^)	13.82 ^a^	13.39 ^b^	13.29 ^b^	13.32 ^b^	13.36 ^b^	13.96 ^a^	0.025	<0.001	<0.001	<0.001
Urinary N (g d^−1^)	3.89	3.62	3.46	3.74	3.64	3.64	0.968	0.368	0.896	0.599
Fecal N (g d^−1^)	4.02 ^b^	4.34 ^a^	4.20 ^ab^	4.15	4.18	4.22	0.176	0.042	0.803	0.607
N deposition (g d^−1^)	5.95 ^a^	5.47 ^ab^	5.38 ^b^	5.68	5.48	5.76	0.584	0.046	0.357	0.192
NPU ** (%)	46.12 ^a^	42.28 ^b^	41.97 ^b^	42.41	44.20	43.04	17.528	0.036	0.916	0.252
BV *** of protein (%)	60.82 ^a^	57.14 ^b^	56.75 ^b^	56.49 ^b^	58.44 ^ab^	60.38 ^a^	15.667	0.015	0.048	0.231

Values with dissimilar lowercase letters in a given row represent a significant difference (*p*< 0.05). Similar letters, or absence of letters, reflect no significant change (*p* > 0.05). VD_3_ * = vitamin D_3_. NPU ** net protein utilization. BV *** = biological value.

**Table 5:** The outcome of Ca and VD_3_ diet supplementation on Ca and P digestibility in maturing and furring blue foxes.

Items	Ca Level (%)			VD_3_ * Level (IU·kg^−1^)			SEM	*p*-Value		
	0	0.4	0.8	1000	2000	4000		Ca Level	VD_3_ Level	Ca × VD_3_ Interaction
Fecal Ca (g·d^−1^)	2.79 ^c^	3.80 ^b^	4.80 ^a^	3.87	3.71	3.76	0.103	<0.001	0.375	0.122
Fecal P (g·d^−1^)	1.36 ^c^	1.88 ^b^	2.54 ^a^	1.92	1.92	1.92	0.015	<0.001	0.704	0.071
Ca digestibility (%)	−4.99	−8.40	−11.65	−9.43	−6.93	−8.56	1.579	0.053	0.699	<0.001
P digestibility (%)	31.50 ^a^	20.89 ^b^	17.60 ^c^	23.90	23.53	22.54	0.922	<0.001	0.740	0.012

Values with dissimilar lowercase letters in a given row represent significant difference (*p* < 0.05). Similar letters, or absence of letters, reflect no significant change (*p* > 0.05). VD_3_ * = vitamin D_3_.

**Table 6:** The outcome of Ca and VD_3_ diet supplementation on serum biochemical indices in maturing and furring blue foxes.

Items	Ca Level (%)			VD_3_ * Level (IU·kg^−1^)			SEM	*p*-Value		
	0	0.4	0.8	1000	2000	4000		Ca Level	VD_3_ Level	Ca × VD_3_ Interaction
TP ** (g L^−1^)	56.32	55.80	54.12	54.03 ^b^	56.47 ^a^	55.84 ^ab^	15.369	0.136	0.008	0.850
Serum Ca (mmol L^−1^)	2.46	2.57	2.50	2.50	2.49	2.55	0.082	0.377	0.741	0.035
Serum P (mmol L^−1^)	1.70	1.72	1.63	1.74	1.67	1.64	0.048	0.292	0.245	0.304
ALP *** (U L^−1^)	53.84	58.40	56.71	56.17	59.08	53.90	1.494	0.446	0.320	0.147

Values with dissimilar lowercase letters in a given row represent a significant difference (*p* < 0.05). Similar letters, or absence of letters, reflect no significant change (*p* > 0.05). VD_3_ * = vitamin D_3_. TP ** = total protein. ALP *** = alkaline phosphatase.

**Table 7:** The outcome of Ca and VD_3_ diet supplementation on serum hormone concentration in maturing and furring blue foxes.

Items	Ca Level (%)			VD_3_ * Level (IU·kg^−1^)			SEM	*p*-Value		
	0	0.4	0.8	1000	2000	4000		Ca Level	VD_3_ Level	Ca × VD_3_ Interaction
PTH ** (pg. ml^−1^)	18.56 ^a^	18.18 ^a^	16.79 ^b^	17.48	17.65	18.50	2.900	0.003	0.072	<0.001
CT *** (pg. ml^−1^)	15.42 ^a^	13.06 ^b^	12.78 ^b^	15.34 ^a^	14.18 ^b^	11.79 ^c^	3.095	<0.001	<0.001	<0.001
25-OH-D_3_ (ng ml^−1^)	8.69	8.62	9.00	8.89 ^a^	8.18 ^b^	9.15 ^a^	0.652	0.361	<0.001	0.038

Values with dissimilar lowercase letters in a given row represent a significant difference (*p* < 0.05). Similar letters, or absence of letters, reflect no significant change (*p* > 0.05). VD_3_ * = vitamin D_3_. PTH ** = parathyroid hormone. CT *** = calcitonin.

## Data Availability

The data presented in this study are available on request. These data are not publicly available to preserve the data privacy of the commercial farm.
